# The effect of systemic hyperthermia on melphalan pharmacokinetics in mice.

**DOI:** 10.1038/bjc.1985.11

**Published:** 1985-01

**Authors:** D. J. Honess, J. Donaldson, P. Workman, N. M. Bleehen

## Abstract

The effect of 45 min systemic heating at 41 degrees C on plasma and RIF-1 tumour pharmacokinetics of intraperitoneally administered melphalan (MEL) was studied in C3H mice. This heat dose causes greater potentiation of MEL in tumour than in marrow cells, resulting in a therapeutic gain for the combined therapy (Honess & Bleehen, 1985). MEL (7.5 mg kg-1) was administered at the start of heating and concentrations assayed from 20-90 min by high-performance liquid chromatography (HPLC). With or without heat peak concentrations were achieved by 20 min and were 3 to 4 micrograms ml-1 in plasma and 1-3 micrograms g-1 in tumour. Higher MEL concentrations in both plasma and tumour were found in heated animals at times after 20 min from injection, but the effect was greater in plasma (2.5-4 fold) than in tumour (1.5-2 fold) where differences were not always significant. At 40 min after a dose of 7.5 mg kg-1, plasma and tumour concentrations in heated animals were equivalent to those after 12.5 mg kg-1 and 8.5 mg kg-1, respectively, without heating. Tumour/plasma ratios were usually lower in heated than in unheated animals where they often exceeded 100%. The apparent plasma elimination half-life (t1/2) was 17.5-25 min in unheated and 24-44 min in heated animals. The area under the curve (AUC) was increased by a factor of 1.2-1.5 in heated animals, at least partly due to a decrease in volume of distribution. The heat induced increase in MEL exposure may be involved in the enhanced response to the drug, but does not appear to explain the therapeutic gain compaired to MEL alone.


*Br. J. Cancer* (1985), **51**, 77–84

### The effect of systemic hyperthermia on melphalan pharmacokinetics in mice

D.J. Honess, J. Donaldson, P. Workman & N.M. Bleehen

*MRC Unit and University Department of Clinical Oncology and Radiotherapeutics, Hills Road, Cambridge CB2 2QH, UK.*

**Summary** The effect of 45 min systemic heating at 41°C on plasma and RIF-1 tumour pharmacokinetics of intraperitoneally administered melphalan (MEL) was studied in C3H mice. This heat dose causes greater potentiation of MEL in tumour than in marrow cells, resulting in a therapeutic gain for the combined therapy (Honess & Bleehen, 1985). MEL (7.5 mg kg$^{-1}$) was administered at the start of heating and concentrations assayed from 20–90 min by high-performance liquid chromatography (HPLC). With or without heat peak concentrations were achieved by 20 min and were 3 to 4 $\mu$g ml$^{-1}$ in plasma and 1–3 $\mu$g g$^{-1}$ in tumour. Higher MEL concentrations in both plasma and tumour were found in heated animals at times after 20 min from injection, but the effect was greater in plasma (2.5–4 fold) than in tumour (1.5–2 fold) where differences were not always significant. At 40 min after a dose of 7.5 mg kg$^{-1}$, plasma and tumour concentrations in heated animals were equivalent to those after 12.5 mg kg$^{-1}$ and 8.5 mg kg$^{-1}$, respectively, without heating. Tumour/plasma ratios were usually lower in heated than in unheated animals where they often exceeded 100%. The apparent plasma elimination half-life ($t\frac{1}{2}$) was 17.5–25 min in unheated and 24–44 min in heated animals. The area under the curve (AUC) was increased by a factor of 1.2–1.5 in heated animals, at least partly due to a decrease in volume of distribution. The heat induced increase in MEL exposure may be involved in the enhanced response to the drug, but does not appear to explain the therapeutic gain compaired to MEL alone.

We have previously shown that potentiation of melphalan (MEL) by 45 min whole body heating at 41°C is greater in two tumours (RIF-1 and KHT) than in normal marrow stem cells (CFUs) in C3H mice (Honess & Bleehen, 1985). This results in a therapeutic gain for the combination of whole body heat with MEL. In contrast, under the same conditions, no gain was found for the combination with 3 other alkylating agents, (chlorambucil, cis-platinum and cyclophosphamide), or the nitrosoureas BCNU and CCNU (Honess & Bleehen, 1982, 1985). One of the possible mechanisms may be alteration of drug pharmacokinetics by heat, a topic on which few studies have been carried out (Mimnaugh *et al.*, 1978; Honess *et al.*, 1980; Magin *et al.*, 1980). We have therefore studied the effect of systemic heat at 41°C on the pharmacokinetrics of MEL in plasma and in the RIF-1 tumour in order to investigate whether any heat induced changes in pharmacokinetics would account for the greater heat potentiation in tumour. We have used a paired-ion reversed-phase high-performance liquid chromatography (HPLC) technique to assay MEL in plasma and tumour in unheated and heated mice.

Correspondence: D.J. Honess.
Received 3 July 1984; and in revised form 9 October 1984.

### Materials and methods

### *Mice*

Female C3H/He mice were obtained from Olac (Southern) Ltd, Bicester, UK. Female C3H/Km mice were bred in this unit. Mice were treated at 20–30 g weight.

### *Tumour*

The RIF-1 tumour described by Twentyman *et al.* (1980) was grown i.m. in the left hind leg, initiated by an inoculum of $10^5$ cells from culture. Animals were treated when tumours reached mean leg diameters of 9–11 mm, usually on Day 12 or 13 after inoculation. These tumours were slightly larger than those used in the work in which heat potentiation of mel was demonstrated (8–9.5 mm diameter, Honess & Bleehen, 1985). This was in order to provide sufficient tumour for MEL assay. All experiments reported in this paper were carried out on tumour-bearing animals.

### *Hyperthermia*

The method of inducing systemic hyperthermia by enclosing animals in an incubator has been previously described (Honess and Bleehen 1982).

© The Macmillan Press, 1985

Rectal temperatures reached 41°±0.2°C within 5 to 10 min of the start of heating. Central tumour temperatures were within 0.2°C of rectal temperature. A standard heat treatment of 45 min at 41°C was used, entailing a total of 50 min in the incubator. Thus for time points up to 45 min, mice were heated for the entire time of exposure to MEL, whereas for later time points, mice were removed from the incubator after the standard treatment and kept at room temperature. Drug was always given immediately before the start of heat.

*Drug*

MEL was kindly supplied by Dr Derry Wilman of the Institute of Cancer Research, Sutton, UK. The drug was dissolved in acid ethanol (5% conc. HCl in 95% ethanol), diluted 1 in 10 with propylene glycol-$K_2HPO_4$ buffer to bring the pH to 7.4, and finally diluted 1:10 with cold Hanks' balanced salt solution for injection. The final concentration of ethanol was thus only 1%. It was made up immediately before use, put on ice and injected within 5 min in a volume of 10 ml kg$^{-1}$. A dose of 7.5 mg kg$^{-1}$ was chosen for the majority of the studies because it is the lowest dose in unheated animals for which a therapeutic gain was found for the RIF-1 tumour and is a dose at which it is possible to measure the tumour response in both unheated and heated animals by growth delay (Honess & Bleehen, 1985).

*Measurement of MEL concentrations*

At the time of sacrifice mice were anaesthetised with ether and bled by cardiac puncture using heparinised syringes. Blood was put on ice and plasmas were prepared as soon as possible (within 1 h) and frozen. Tumours were excised as rapidly as possible and snap frozen immediately at −70°C. They were stored at −20°C, then quickly thawed and homogenised with 2 vol of cold water before assay.

MEL was assayed by a paired-ion reversed phase high-performance liquid chromatography technique similar to that of Hinchliffe *et al.* (1983). Full details will be given elsewhere (Lee & Workman, in preparation). Briefly, samples were deproteinized with 4 vol acetonitrile containing 1% v/v HCl. The supernatant was removed and taken to dryness and redissolved in running buffer. The column used was a $\mu$ Bondapak C18 Radial-Pak cartridge (8 mm i.d.; 10 $\mu$m particle size; Waters Associates) and the running buffer was 35% acetonitrile/water containing 5 mM pentane sulphonic acid and 10 mM dibutylamine, adjusted to pH 3 with HCl. The flow rate was 4 ml min$^{-1}$ and absorbance was monitored at 254 mM. Concentrations were obtained from peak heights with reference to linear calibration curves.

*Measurement of MEL hydrolysis* in vitro

Rates of MEL hydrolysis were measured in 0.2 M phosphate buffer pH 7.4 or in 1:1 buffer:plasma at 37°C and 41°C. MEL was spiked at 10 $\mu$g ml$^{-1}$ into thermally equilibrated solutions kept in circulating water baths at 37°C±0.1°C and 41°±0.1°C (Grant Ltd., UK). Samples were taken for HPLC assay at intervals up to 6 h. The pH was checked at the end of the experiments and confirmed still to be 7.4.

*Estimation of pharmacokinetic parameters*

Post-peak plasma concentrations declined approximately exponentially. Plots of log plasma drug concentration against time were fitted by a least squares linear regression analysis, which yielded plasma elimination half lives ($t\frac{1}{2}$) with confidence limits. *In vitro* hydrolysis was also exponential and the same method was used to calculate *in vitro* $t\frac{1}{2}$ values.

The area under the concentration x time curve (AUC) was estimated using a trapezoidal rule modification of Simpson's rule, taking the geometric mean of measured drug concentrations at each time point.

Analysis of the significance of differences between MEL concentrations in heated and unheated animals was carried out using the Wilcoxon Mann-Whitney test. Differences in elimination half-lives were compared using students' t distribution.

### Results

Preliminary experiments with 7.5 mg kg$^{-1}$ MEL given intraperitoneally at the start of heating showed a broad peak of MEL concentration from 10–20 min after injection, which was unchanged by heat. However at times after 20 min higher concentrations were seen in both plasma and tumour of heated animals. In 4 subsequent experiments, with larger numbers of anumals per group (4 and 5), MEL concentrations were measured only at 20, 40, 60 and 90 min after drug administration. At later times the concentrations of drug fell very close to or below the limit of detection (~0.01 $\mu$g ml$^{-1}$).

Results for 2 experiments with i.p. administered MEL are shown in Figure 1 and they illustrate the *greatest* and *smallest* effects of heat observed in this series of 4 experiments. Peak plasma concentrations of 3–4 $\mu$g ml$^{-1}$ were found at 20 min, and no difference was seen in heated animals. In Experiment A plasma concentrations (panel a) were

**Figure 1** Effect of heat (45 min at 41°C) on the plasma and tumour pharmacokinetics of 7.5 mg kg$^{-1}$ melphalan given i.p. at the start of heating. Closed symbols indicate unheated animals, open symbols heated ones. Each point represents one animal. Panels (a) and (b) show data for Expt A, and panels (c) and (d) show data for Expt B. Panels (a) and (c) show plasma data, panels (b) and (d) show the corresponding tumour data. Lines are best fit by eye.

higher ($P \leq 0.025$) in heated animals at 40 min by a factor of up to 4 and remained about 3 times higher ($P \leq 0.005$) for the rest of the measured time course. In Experiment B (panel c) complete separation between the heated an unheated groups was seen only at 60 min ($P \leq 0.005$), although it is clear that plasma MEL concentrations were generally higher in the heated animals by about a factor of 2 ($P \leq 0.005$ at 40 min, $P \leq 0.05$ at 90 min). The MEL concentrations in the tumour (Figure 1, panels b and d) tend to follow those in the plasma; peak concentrations from 1 to 3 $\mu$g ml$^{-1}$ were found at 20 min. In Experiment A which showed the larger increase in plasma concentrations in heated animals, the clear difference between heated and unheated tumours seen at 40 and 60 min ($P<0.001$, panel b) is less obvious at 90 min ($P<0.05$), and MEL concentrations are only 1.5 to 2 times higher in heated tumours. In Experiment B, with a smaller increase in plasma concentrations in heated animals, tumour concentrations are lower than in Experiment A, and there is no significant difference between concentrations in heated and unheated tumours. Nevertheless median values for heated tumours are consistently 1.5–2 times higher than for unheated tumours from 20 to 60 min, as in Experiment A.

The data in Figure 1 therefore indicate that systemic heat at 41°C causes higher plasma and tumour MEL concentrations than are found in unheated animals, but the effect is greater in plasma than in tumour.

Tumour/plasma ratios for both experiments shown in Figure 1 are presented in Table I. The main feature of these data, confirmed in other experiments, is that tumour/plasma ratios are lower in heated animals. With the exception of the 60 min for Experiment B, tumour/plasma ratios measured from 40 to 90 min were 1.5 to 2 times higher in unheated mice. Tumour/plasma ratios greater than 100% were found in Experiment A and in other experiments (not shown).

It seemed possible that the effects shown in Figure 1 might be due to the effect of heat on MEL uptake from the peritoneum. We therefore repeated the experiment giving MEL i.v. (Experiment C) and the results for plasma are shown in Figure 2. Plasma MEL concentrations were again higher in heated than in unheated animals by a factor of 1.5–2, and the results, show the same trend as those shown in Figure 1. This indicates that the increased MEL concentrations in heated animals cannot be attributed to the effect of heat on uptake from the peritoneum.

**Table I** Medians and ranges of tumour/plasma ratios in unheated and heated animals following 7.5 mg kg$^{-1}$ MEL i.p. immediately before the start of heat ($n=4$ or 5)

| | *Tumour/plasma ratio %* | | | |
|---|---|---|---|---|
| | *Expt A* | | *Expt B* | |
| *Time after MEL (min)* | *Unheated* | *Heated* | *Unheated* | *Heated* |
| 20 | 78 (44–121) | 68 (62–128) | 31 (25–47) | 53 (45–61) |
| 40 | 154 (129–176) | 104 (92–116) | 62 (32–82) | 36 (28–91) |
| 60 | 143 (101–170) | 95 (62–131) | 40 (10–58) | 38 (21–60) |
| 90 | 100 (56–113) | 50 (32–59) | 77 (33–96) | 40 (22–55) |

**Figure 2** Effect of heat (45 min at 41°C) on the plasma pharnacokinetics of 7.5 mg kg$^{-1}$ melphalan given i.v. at the start of heating. Closed symbols represent unheated animals, open symbols represent heated animals. Lines are best fit by eye.

The apparent plasma t½ values for MEL in heated and unheated animals for Experiments A, B and C are presented in Table II. For the i.p. route in unheated animals the t½ values of around 20 min are in good agreement with previously published data (e.g. Hinchliffe *et al.*, 1983) and are also very similar to that for the i.v. route. In heated animals, the calculated t½ values are significantly greater than in unheated ones, but considering the shapes of the elimination curves in Figure 1 (panels a and

**Table II** Apparent t½ values (min) for MEL in unheated and heated animals treated with 7.5 mg kg$^{-1}$ MEL

| *Expt.* | *Unheated* | *Heated* |
|---|---|---|
| A Expt. (i.p.) | 25 (20–32) | 44 (34–60) $0.01>P>0.001$ |
| B Expt. B (i.p.) | 17.5 (15–21) | 24 (21–28) $0.01>P>0.001$ |
| C Expt. (i.v.) | 19 (16–23) | 18 (16–21) $P>0.1$ |

Values in brackets are 95% confidence limits c) over only 4 time points, it is difficult to tell whether there is a true lengthening of t½, or alternatively whether the peak is being maintained for rather longer. Any peak-broadening in the plasma elimination curves did not appear sufficiently marked to justify excluding data points from the t½ estimation. However, it is clear that the lines joining median concentrations tended to be parallel after 40 min in all experiments. Moreover, Table II shows that there was no change in elimination t½ when MEL was given i.v. Taken together these data suggest that the elevated plasma and tumour concentrations probably result from a heat-induced reduction in the volume of MEL distribution. Heated animals suffered a weight loss of 3, 5 and 7% at 20, 40 and 50 min respectively after the start of heating, which was rapidly regained at normal temperature. This fluid loss provides a plausible physiological explanation for the reduced volume of MEL distribution.

It is clearly that the calculated plasma t½ values in heated animals are certainly no shorter than in unheated animals. This is in contrast to *in vitro* results for MEL hydrolysis. Data presented in Table III show that *in vitro* hydrolysis proceeds more slowly in plasma than in buffer, but that in both environments the t½ is shorter at 41°C by a factor of 1.5 to 1.9. The values for MEL in buffer at 37°C are in good agreement with the previously published values of 58 min and 60 min obtained by

**Table III** t½ values for MEL in 0.2 M phosphate buffer pH 7.4 or 1:1 plasma:buffer pH 7.4, measured at 37°C and 41°C *in vitro*.

| | | *t½ 37°C (min)* | *t½ 41°C (min)* | *t½ 37°C/t½ 41°C* |
|---|---|---|---|---|
| Expt. 1 | Buffer | 58 (54–63) | 31 (29–33) | 1.9 |
| | Plasma:buffer (1:1) | 93 (83–106) | 56 (49–66) | 1.7 |
| Expt. II | Buffer | 45 (40–52) | 30 (27–34) | 1.5 |
| | Plasma:buffer (1:1) | 82 (73–94) | 54 (40–58) | 1.5 |

Values in brackets give 95% confidence limits estimation of residual alkylating activity using the Epstein reagent or by titration of acid release respectively (Workman *et al.*, 1976).

A widely used index of drug exposure is the area under the concentration × time curve (AUC). AUCs have been calculated for plasma and tumour in unheated and heated animals for experiments A, B and C and are presented in Table IV. The change in plasma AUC in heated animals is similar to the change in tumour AUC, an increase by a factor of 1.2 to 1.5. The changes in AUC brought about by heat depend on the precise shapes of the control and heat curves in each particular case. In Experiment A the increase in plasma AUC was slightly greater than in tumour whereas in Experiment B this small difference was reversed. We conclude that in terms of AUC, heat causes no consistent elevation in tumour compared to plasma MEL exposure.

We wished to obtain some estimate of the MEL dose in unheated animals which would give MEL concentrations similar to those measured in heated animals after 7.5 mg kg$^{-1}$ i.p. MEL (i.e. an iso-effect dose), and thus to derive some alternative measure of drug exposure modification by heat. On the basis of Experiment A and another (not shown) the 40 min time point was selected as that when the difference between plasma concentrations in heated and unheated animals was greatest, and so in experiments B and C we also measured MEL concentrations for a range of drug doses in unheated animals at 40 min. The data are shown in Figure 3. Lines of best fit have been drawn through the data by eye for unheated animals, and hence the iso-effect dose was estimated. For plasma in Experiment B (i.p. administration, panel a) the value is 12.5 mg kg$^{-1}$ and in Experiment C (i.v. administration, panel c) the value is 13.0 mg kg$^{-1}$.

**Table IV** $AUC_{0-90}$ min values for plasma and tumour in unheated and heated animals

| | *Plasma* | | | *Tumour* | | |
|---|---|---|---|---|---|---|
| *Expt* | *Unheated* $\mu g\ min\ ml^{-1}$ | *Heated* $\mu g\ min\ ml^{-1}$ | *Heated/ unheated* | *Unheated* $\mu g\ min\ g^{-1}$ | *Heated* $\mu g\ min\ g^{-1}$ | *Heated/ unheated* |
| A (i.p.) | 110 | 169 | 1.5 | 112 | 157 | 1.3 |
| B (i.p.) | 122 | 152 | 1.2 | 48 | 69 | 1.5 |
| C (i.v.) | 98 | 126 | 1.3 | n.d. | n.d. | n.d. |

n.d. not determined

**Figure 3** Geometric mean ($n = 5$) plasma or tumour MEL concentrations at 40 min after drug administration, plotted against dose of drug given. Panel a shows plasma data for Expt B and panel b shows tumour data for Expt B i.e. drug given i.p. Panel c shows plasma data for Expt C i.e. drug given i.v. Closed symbols indicate data for unheated animals, open symbols for heated ones. Lines are best fit by eye.

Thus the dose of MEL in unheated animals giving the same mean plasma MEL concentrations at 40 min as 7.5 mg kg$^{-1}$ in heated animals is larger by a factor of ~1.7. However, for the tumour in Experiment B, (panel b) the iso-effect dose is 8.5 mg kg$^{-1}$, i.e. it is larger by a factor of only 1.1.

### Discussion

There have been few studies to date on the effect of heat, systemic or local, on drug pharmacokinetics. Effects of local heating on pharmacokinetics in mice under conditions of drug potentiation have been reported by Honess *et al.* (1980) for MISO and by Magin *et al.* (1980) for adriamycin. No change in plasma clearance was found for either drug, but evidence for increased drug metabolism in heated tumours was found for adriamycin. The effects of systemic heat at 42.3°C on adriamycin in non-tumour bearing rabbits were reported by Mimnaugh *et al.* (1978) and they found no difference in plasma clearance. A number of groups have measured drug concentrations in heated tumours under conditions of potentiation and some report increased uptake or binding for cis-platinum in mice (Alberts *et al.*, 1980) and nitrogen mustard in rabbits (Shingleton, 1962). Reduced concentrations in mouse tumours have been reported for misonidazole (Honess et al., 1980) and cyclophosphamide and thiotepa (Longo *et al.*, 1983), also under conditions of drug potentiation. Osieka *et al.* (1978) found no difference in methyl-CCNU uptake in human colon xenografts in nude mice.

In this study we have shown that systemic heat at 41°C for 45 min causes an increase in both plasma and tumour concentrations of MEL, but there is no evidence for a greater elevation in tumour compared to plasma.

In general, tumour/plasma ratios were higher in unheated animals than in heated ones. Nonetheless, the time course of the rise and fall of tumour/plasma ratios, peaking 40–60 min after drug administration, was similar in both heated and unheated animals. Tumour/plasma ratios greater than 100% were found in both heated and unheated tumours; however, the highest values were found in unheated tumours, as were a larger proportion of high values. The accumulation of MEL in murine leukaemia and human breast tumour cells at normal temperature *in vitro* occurs by means of two distinct amino acid carriers (Vistica, 1979, Begleiter *et al.*, 1979, 1980). Our data can be interpreted as evidence for the accumulation of MEL in a solid tumour, with the additional suggestion that heat might impair the functioning of the carrier systems. These conclusions must necessarily be very tentative, since we have only been able to measure average MEL concentrations throughout the homogenised bulk of the tumour. We have no information on intracellular and extracellular concentrations of MEL, which may differ substantially from one another. Nonetheless, Begleiter *et al.* (1980) quote cell:medium ratios of about 3.5 in MCF-7 breast cancer cells in the presence of 10 $\mu$M $^{14}$C melphalan, which is ~3 $\mu$g ml$^{-1}$. We found tumour/plasma ratios of up to 1.7 following peak plasma concentrations of about 3 $\mu$g ml$^{-1}$ (Figure 1, Table I) and have also measured tumour/plasma ratios well in excess of 2 following plasma concentrations of about 6 $\mu$g ml$^{-1}$ 40 min after a dose of 20 mg kg$^{-1}$ MEL (data not shown). It therefore seems possible that the accumulation described *in vitro* may be attained *in vivo*.

The elimination $t\frac{1}{2}$ of MEL was found to be no shorter in heated than unheated animals. One might have expected a shorter $t\frac{1}{2}$ in heated animals, due to the more rapid hydrolysis at 41°C which we demonstrated *in vitro* (Table III). This disparity might be because the hydrolysis contribution to MEL elimination is relatively small, the $t\frac{1}{2}$ value for MEL in 1:1 plasma:buffer *in vitro* being 80 or 90 min at 37°C compared with an elimination $t\frac{1}{2}$ in the mouse of ~20 min. Alternatively it is possible that heat in some way tends to lengthen the elimination $t\frac{1}{2}$ of MEL, and that this is almost compensated for by the increased rate of hydrolysis. While this method of heating has been shown to impair glomerular filtration. (M.I. Walton, personal communication) it is unlikely that this would greatly affect MEL clearance since it has been reported that <2% of the injected dose was excreted as MEL in mouse urine (Furner *et al.*, 1976). It has been shown that MEL does not undergo important metabolism (Evans *et al.*, 1982) but that a major route of elimination of MEL in all species examined is biliary excretion, (Furner & Brown, 1980). We have no information on the effect of this method of systemic heating on biliary excretion. Another important aspect of MEL pharmacology is its binding to plasma proteins (Ehrsson & Lonroth, 1982) which is ~96% in mice (F.Y.F. Lee, personal communication). It is not possible to determine the effect of heat on protein binding directly *in vivo*, and in the present work we have determined total (bound plus free) concentrations. Since changes in protein binding could affect the amount of MEL available for uptake into cells, we are currently developing techniques to study the effect of heat on protein binding *in vitro*.

The data for intravenously administered MEL (Figure 2), also showing an increase in plasma MEL concentration, indicated that the effect of heat was not primarily on MEL uptake from the peritoneum. The higher peak MEL concentration in heated animals and unchanged $t\frac{1}{2}$ were compatible with a probable reduction of apparent volume of distribution of the MEL in heated mice. This would be consistent with the weight loss observed during heating.

It is interesting that in two experiments (one i.p., one i.v.) heating produced mean MEL concentrations 40 min after administration of 7.5 mg kg$^{-1}$ equivalent to those produced by ~1.7 × that dose in unheated animals (Figure 3). For bone marrow stem cells, 12.5 mg kg$^{-1}$ MEL in unheated animals in equitoxic with 7.3 mg kg$^{-1}$ in heated animals (Honess & Bleehen, 1985), also a ratio of 1.7. If toxicity of MEL to bone marrow stem cells is closely related to MEL plasma concentration, as would seem possible, then the effect of heat on MEL plasma concentration would be sufficient to account for the heat potentiation of MEL toxicity in CFUs. However, in RIF-1 tumour 12.5 mg kg$^{-1}$ MEL in unheated animals is equitoxic with only 4.6 mg kg$^{-1}$ MEL in heated animals (Honess & Bleehen, 1985), a ratio of 2.7. This ratio is substantially larger than the ratio of doses resulting in equal MEL plasma concentrations at 40 min in unheated and heated animals (1.7, Figure 3, panel a). It is also very much greater than the ratio of doses resulting in equal tumour concentrations at 40 min in unheated and heated animals, which is 1.1 (Figure 3, panel b). Although the small increase in MEL concentration in heated tumours presumably contributes to the MEL potentiation, it would appear to be only a small component of the mechanism.

While it seems reasonable to compare changes in tumour pharmacokinetics with changes in drug toxicity in the tumour under the same conditions, it is less satisfactory to compare changes in plasma pharmacokinetics with changes in drug toxicity to the marrow. Although the marrow is well perfused, MEL access to cells requires carrier mediation, and the behaviour of such carriers in CFUs, especially under heated conditions, cannot be easily predicted. It would therefore be preferable to monitor MEL pharmacokinetics in the CFUs. However since the CFUs comprise only 0.015% of the nucleated cells of the bone marrow, and are only functionally identificable, this is clearly impossible. Nonetheless there is a good correlation between increase in plasma MEL concentration and toxicity to CFUs.

The broad conclusion from this study is that systemic heat at 41°C does increase plasma and tumour MEL concentrations. However while changes in plasma pharmacokinetics may account for the increase in MEL toxicity to marrow, the changes in tumour pharmacokinetics can only contritute in a minor way to the greater heat potentiation of MEL in tumour. These results therefore do not explain the therapeutic gain found for this combination of heat and MEL, and the reason for this probably lies at the cellular biochemical level.

### References

ALBERTS, D.S., PENG, Y.M., CHEN, G., MOON, T.E., CETAS, T.C. & HOESCHELE, J.D. (1980). Therapeutic synergism of hyperthermia-cis-platinum in a mouse tumour model. *J. Natl Cancer Inst.*, **65**, 455.

BEGLEITER, A., FROESE, E.K. & GOLDENBERG, G.J. (1980). A comparison of melphalan transport in human breast cancer cells and lymphocytes *in vitro*. *Cancer Letters*, **10**, 243.

BEGLEITER, A., LAM, H.-Y.P., GROVER, J., FROESE, E. & GOLDENBERG, G.J. (1979). Evidence for active transport of melphalan by two amino acid carriers in L 5178Y lymphoblasts in vitro. *Cancer Res.*, **39**, 353.

EHRSSON, H. & LONROTH, U. (1982). Degradation of melphalan in aqueous solutions – influence of human albumin binding. *J. Pharmacol. Sci.*, **71**, 826.

EVANS, T.L., CHANG, S.Y., ALBERTS, D.S., SIPES, I.G. & BRENDEL, K. (1982). In vitro degradation of L-phenyl alanine mustard (L-PAM). *Cancer Chemother. Pharmacol.*, **8**, 175.

FURNER, R.L. & BROWN, R.K. (1980). L-phenyl alanine mustard (L-PAM): the first 25 years. *Cancer Treat. Reps.*, **64**, 559.

FURNER, R.L., MELLETT, L.B., BROWN, R.K. & DUNCAN, G. (1976). A method for the measurement of L-phenyl alanine mustard in the dog and mouse by high pressure liquid chromatography. *Drug. Metab. Dispos.*, **4**, 577.

HINCHLIFFE, M., McNALLY, N.J. & STRATFORD, M.R.L. (1983). The effect of radiosensitisers on the pharmacokinetics of melphalan and cyclophosphamide in the mouse. *Br. J. Cancer*, **48**, 375.

HONESS, D.J. & BLEEHEN, N.M. (1982). Sensitivity of normal mouse marrow and RIF-1 tumour to hyperthermia combined with cyclophosphamide or BCNU: a lack of therapeutic gain. *Br. J. Cancer*, **46**, 236.

HONESS, D.J. & BLEEHEN, N.M. (1985). Thermo-chemotherapy with cis-platinum, CCNU, BCNU, chlorambucil and melphalan on murine marrow and 2 tumours; therapeutic gain for melphalan only. *Br. J. Radiol.*, **58**, 63.

HONESS, D.J., WORKMAN, P., MORGAN, J.E. & BLEEHEN, N.M. (1980). Effects of local hyperthermia on the pharmacokinetics of misonidazole in the anaesthetised mouse. *Br. J. Cancer*, **41**, 529.

LONGO, F.W., TOMASHEFSKY, P., RIVIN, B.D. & TANNENBAUM, M. (1983). Interaction of ultrasonic hyperthermia with two alkylating agents in a murine bladder tumour. *Cancer Res.*, **43**, 3231.

MAGIN, R.L., CYSYK, R.L. & LITTERST, C.L. (1980). Distribution of adriamycin in mice under conditions of local hyperthermia which improve systemic drug therapy. *Cancer Treat. Rep.*, **64**, 203.

MIMNAUGH, E.G., WARING, R.W., SIKIC, B.I. & 5 others (1978). Effect of whole body hyperthermia on the disposition and metabolism of adriamycin in rabbits. *Cancer Res.*, **38**, 1420.

OSIEKA, R., MAGIN, R.L. & ATKINSON, E.T. (1978). The effect of hyperthermia on human colon cancer xenografts in nude mice. In: *Proceedings of the 2nd International Symposium on Cancer Therapy by Hyperthermia and Radiation*, Essen, June 1977. Urban & Schwartzenberg, Baltimore, p. 287.

SHINGLETON, W.W., BRYAN, F.A., O'QUINN, W.L. & KRUEGER, L.C. (1962). Selective heating and cooling of tissue in cancer chemotherapy. *Ann. Surg.*, **156**, 406.

TWENTYMAN, P.R., BROWN, J.M., GRAY, J.W., FRANKO, A.J., SCOLES, M.A. & KALLMAN, R.F. (1980). A new mouse model tumour system (RIF-1) for comparison of endpoint studies. *J. Natl Cancer Inst.*, **64**, 595.

VISTICA, D.T. (1979). Cytotoxicity as an indicator for transport mechanisms: evidence that melphalan is transported by two leucine-preferring carrier systems in the L1210 murine leukaemia cell. *Biochim. Biophys. Acta*, **550**, 309.

WORKMAN, P., DOUBLE, J.A. & WILMAN, D.E.V. (1976). Enzyme activated anti-tumour agents-III. Hydrolysis of conjugates of *p*-hydroxyaniline mustard in aqueous solution. *Biochem. Pharmacol.*, **25**, 2347.


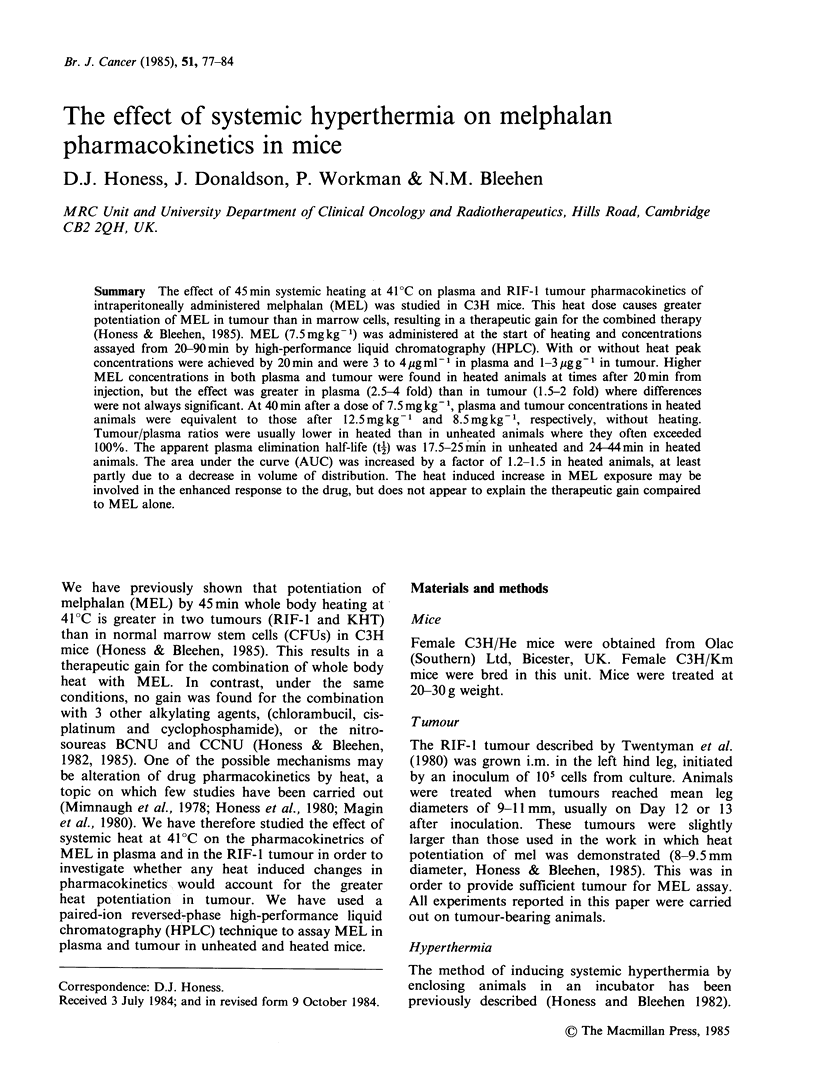

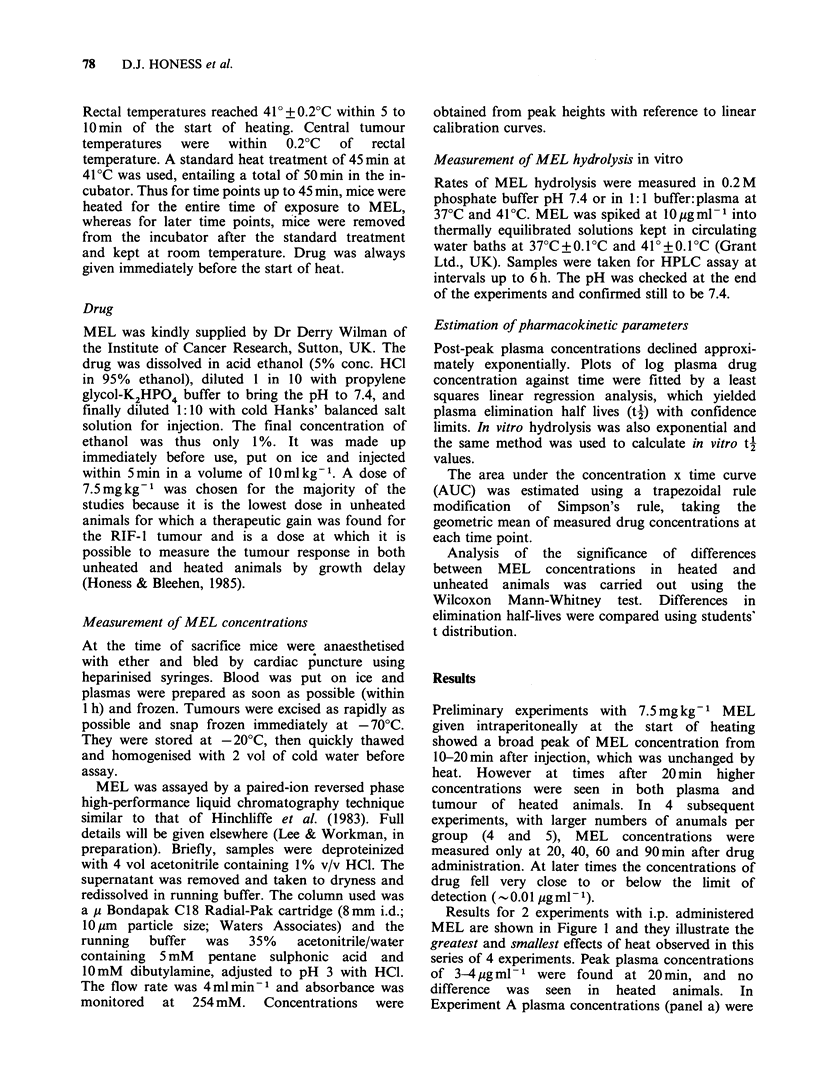

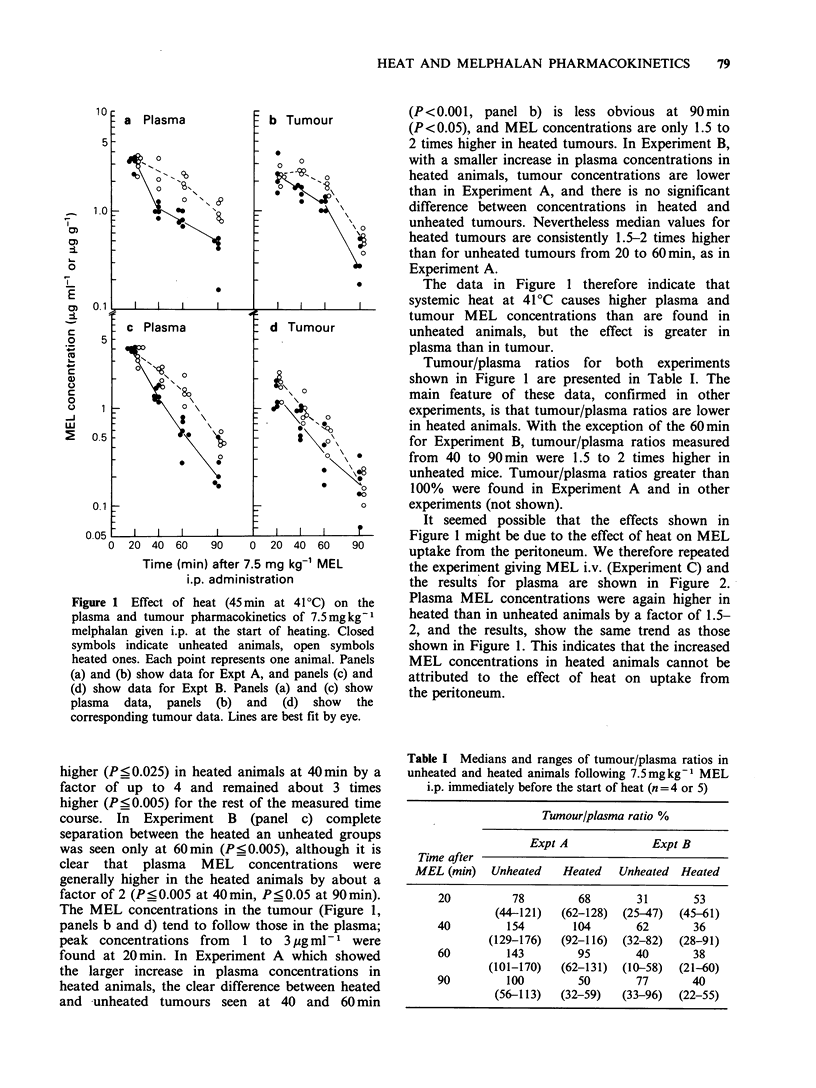

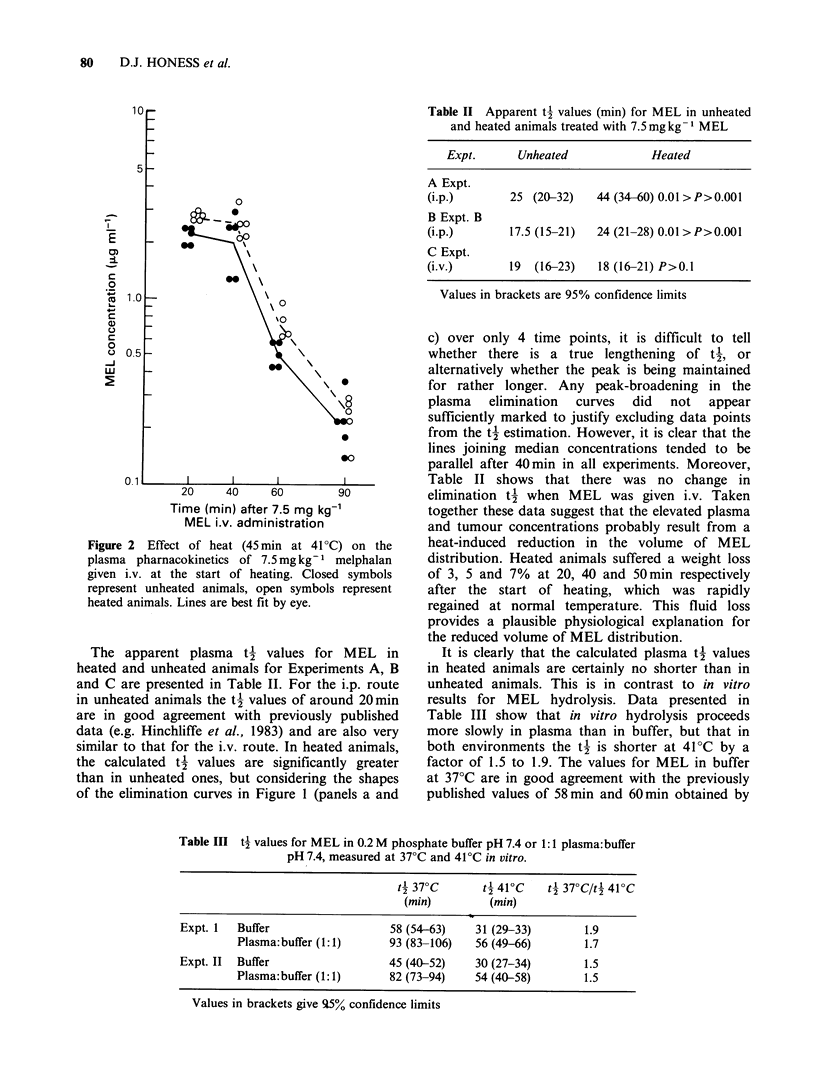

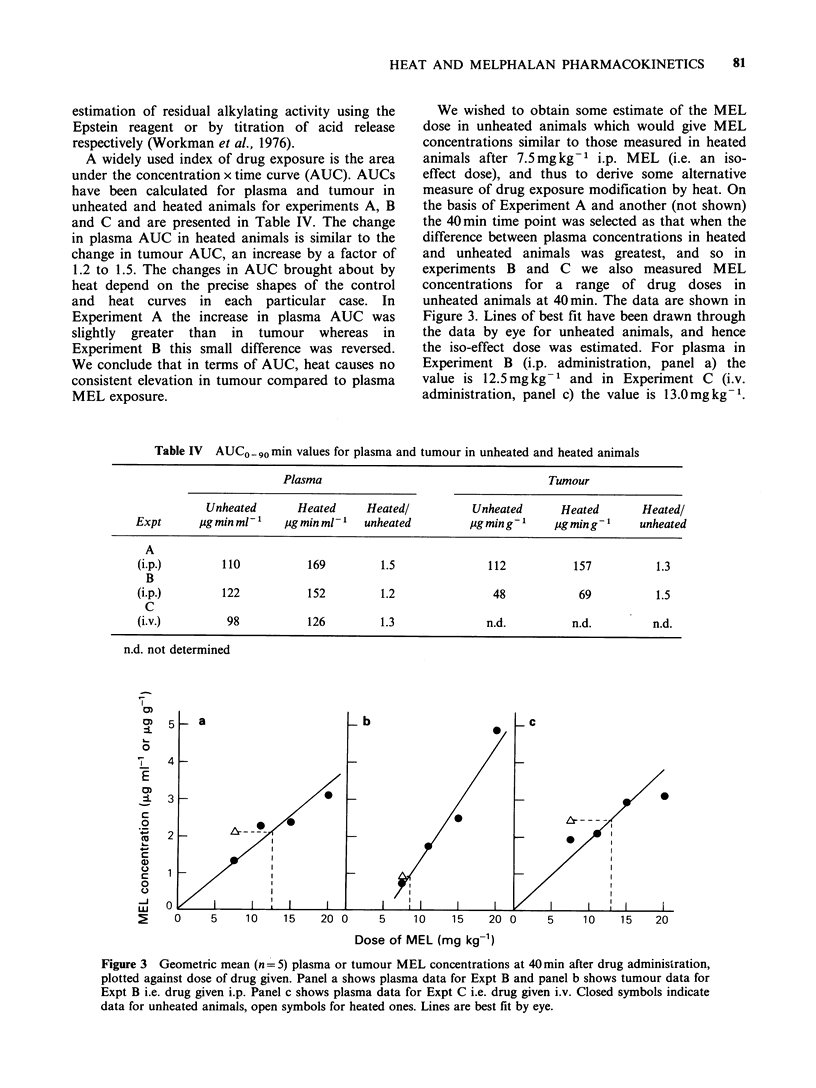

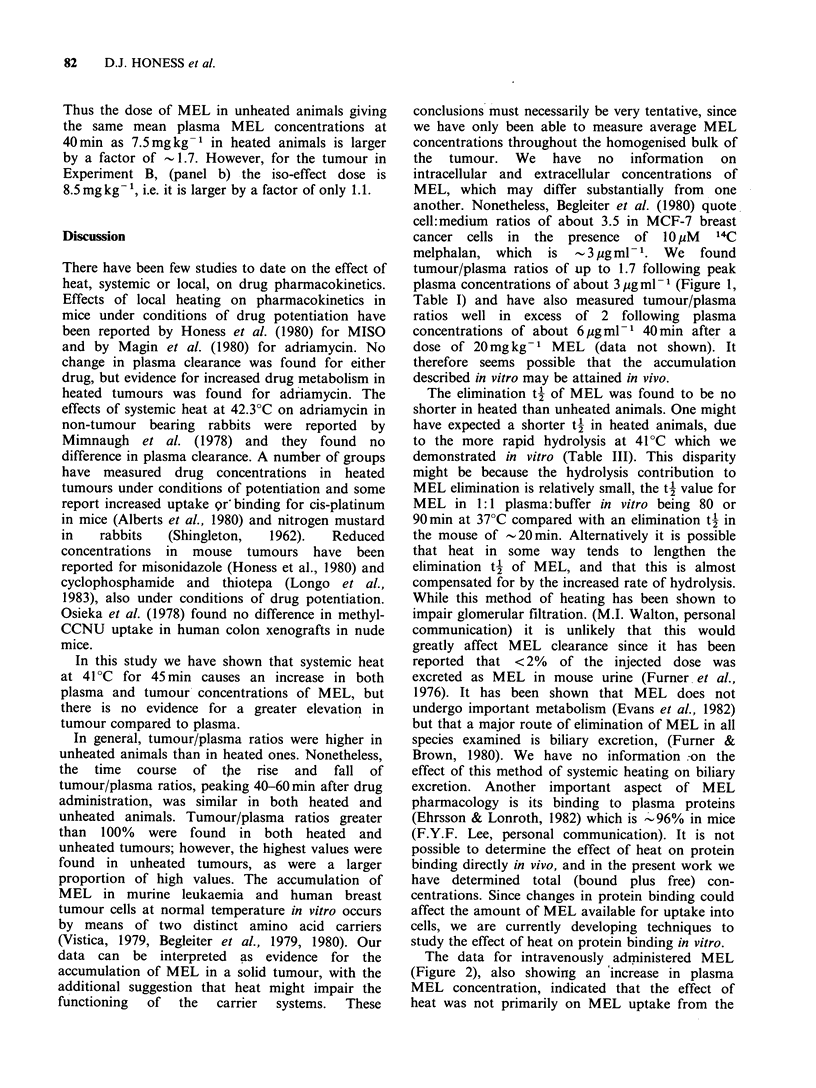

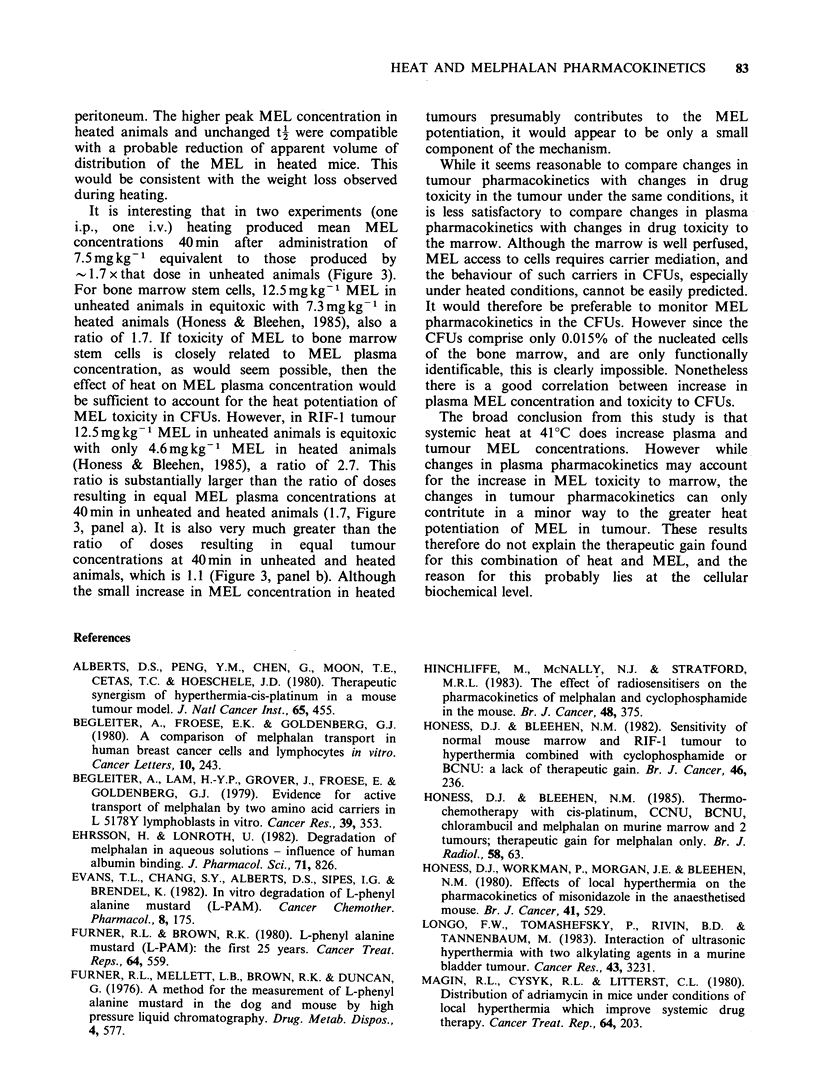

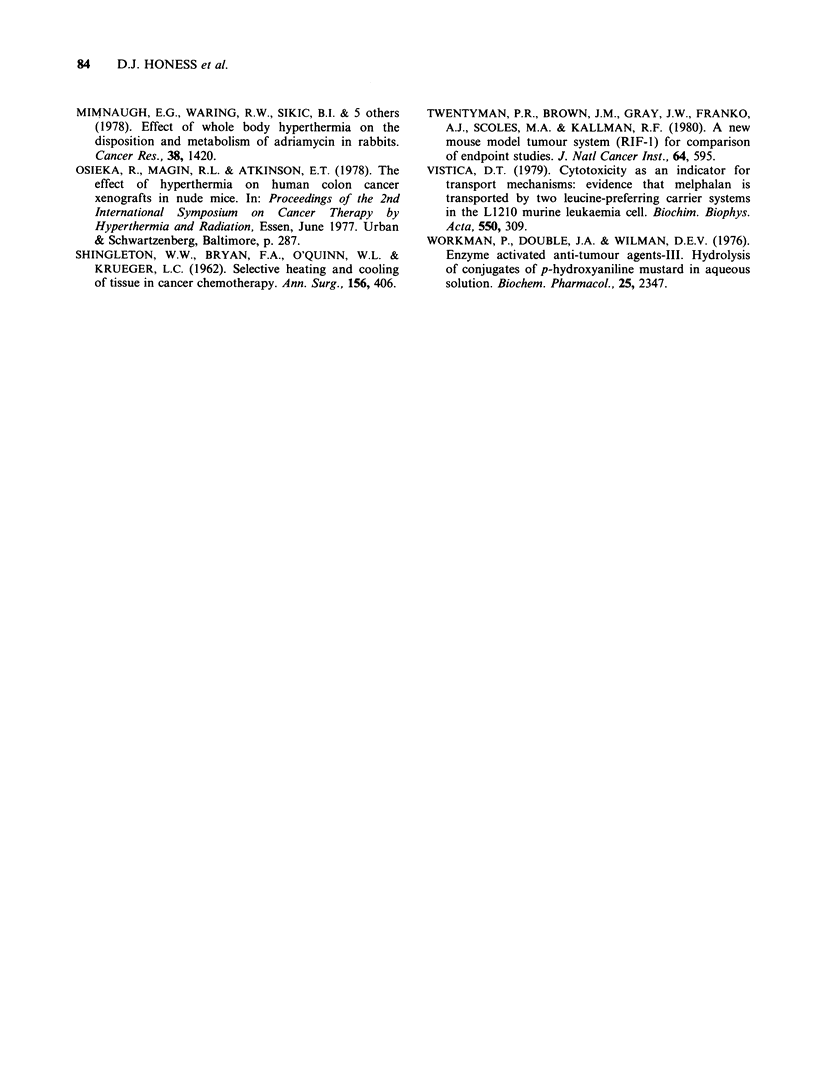


## References

[OCR_00823] Alberts D. S., Peng Y. M., Chen H. S., Moon T. E., Cetas T. C., Hoeschele J. D. (1980). Therapeutic synergism of hyperthermia-cis-platinum in a mouse tumor model.. J Natl Cancer Inst.

[OCR_00829] Begleiter A., Froese E. K., Goldenberg G. J. (1980). A comparison of melphalan transport in human breast cancer cells and lymphocytes in vitro.. Cancer Lett.

[OCR_00835] Begleiter A., Lam H. Y., Grover J., Froese E., Goldenberg G. J. (1979). Evidence for active transport of melphalan by two amino acid carriers in L5178Y lymphoblasts in vitro.. Cancer Res.

[OCR_00841] Ehrsson H., Lönroth U. (1982). Degradation of melphalan in aqueous solutions--influence of human albumin binding.. J Pharm Sci.

[OCR_00846] Evans T. L., Chang S. Y., Alberts D. S., Sipes I. G., Brendel K. (1982). In vitro degradation of L-phenylalanine mustard (L-PAM).. Cancer Chemother Pharmacol.

[OCR_00852] Furner R. L., Brown R. K. (1980). L-phenylalanine mustard (L-PAM): the first 25 years.. Cancer Treat Rep.

[OCR_00857] Furner R. L., Mellett L. B., Brown R. K., Duncan G. (1976). A method for the measurement of L-phenylalanine mustard in the mouse and dog by high-pressure liquid chromatography.. Drug Metab Dispos.

[OCR_00864] Hinchliffe M., McNally N. J., Stratford M. R. (1983). The effect of radiosensitizers on the pharmacokinetics of melphalan and cyclophosphamide in the mouse.. Br J Cancer.

[OCR_00870] Honess D. J., Bleehen N. M. (1982). Sensitivity of normal mouse marrow and RIF-1 tumour to hyperthermia combined with cyclophosphamide or BCNU: a lack of therapeutic gain.. Br J Cancer.

[OCR_00877] Honess D. J., Bleehen N. M. (1985). Thermochemotherapy with cis-platinum, CCNU, BCNU, chlorambucil and melphalan on murine marrow and two tumours: therapeutic gain for melphalan only.. Br J Radiol.

[OCR_00884] Honess D. J., Workman P., Morgan J. E., Bleehen N. M. (1980). Effects of local hyperthermia on the pharmacokinetics of misonidazole in the anaesthetized mouse.. Br J Cancer.

[OCR_00890] Longo F. W., Tomashefsky P., Rivin B. D., Tannenbaum M. (1983). Interaction of ultrasonic hyperthermia with two alkylating agents in a murine bladder tumor.. Cancer Res.

[OCR_00896] Magin R. L., Cysyk R. L., Litterst C. L. (1980). Distribution of adriamycin in mice under conditions of local hyperthermia which improve systemic drug therapy.. Cancer Treat Rep.

[OCR_00904] Mimnaugh E. G., Waring R. W., Sikic B. I., Magin R. L., Drew R., Litterst C. L., Gram T. E., Guarino A. M. (1978). Effect of whole-body hyperthermia on the disposition and metabolism of adriamycin in rabbits.. Cancer Res.

[OCR_00923] Twentyman P. R., Brown J. M., Gray J. W., Franko A. J., Scoles M. A., Kallman R. F. (1980). A new mouse tumor model system (RIF-1) for comparison of end-point studies.. J Natl Cancer Inst.

[OCR_00929] Vistica D. T. (1979). Cytotoxicity as an indicator for transport mechanism: evidence that melphalan is transported by two leucine-preferring carrier systems in the L1210 murine leukemia cell.. Biochim Biophys Acta.

[OCR_00936] Workman P., Double J. A., Wilman D. E. (1976). Enzyme activated anti-tumour agents--III. Hydrolysis of conjugates of p-hydroxyaniline mustard in aqueous solution.. Biochem Pharmacol.

